# Spinal Microglial TLR7 Activation Drives Hyperalgesia in a Lupus Mouse Model via Upregulation of IL-1β, IL-18, and Cav2.2 and Enhanced Glutamatergic Synaptic Activity

**DOI:** 10.3390/cells15010020

**Published:** 2025-12-22

**Authors:** Saumya Bipin, Viacheslav Viatchenko-Karpinski, Catherine Li, Sujin Lim, Han-Rong Weng

**Affiliations:** 1 Department of Basic Sciences, California Northstate University College of Medicine, 9700 W Taron Drive, Elk Grove, CA 95757, USA; saumya.bipin@cnsu.edu (S.B.); catherine.li11708@cnsu.edu (C.L.); sujin.lim11464@cnsu.edu (S.L.); 2 Department of Biomedical Sciences, Mercer University School of Medicine, Macon, GA 31207, USA; viatc001@umn.edu

**Keywords:** nociception, neuroinflammation, glial neuronal interaction, synaptic, EPSC

## Abstract

**Highlights:**

**What is the main finding?**

- Patients with systemic lupus erythematosus (SLE) often suffer from chronic pain due to the insufficient efficacy and safety profile of currently available analgesics. In this study, we revealed that TLR7 signaling activity is elevated in the spinal dorsal horn in lupus mice with thermal hyperalgesia. TLR7 activation drives molecular, synaptic, cellular, and pain phenotype alterations in lupus mice.

**What is the implication of the main finding?**

- Our findings suggest that targeting TLR7 or downstream effectors may represent a promising strategy to alleviate chronic pain induced by SLE.

**Abstract:**

Patients with systemic lupus erythematosus (SLE) often suffer from chronic pain due to a lack of effective and safe analgesics. In this study, we investigated the role of spinal TLR7 in the pathogenesis of chronic pain using female *MRL* lupus prone (*MRL/lpr*) mice, a SLE mouse model. We found that from 11 weeks of age, *MRL/lpr* mice exhibited thermal hypersensitivity in the hind paw, which reached plateau between 14 and 16 weeks. *MRL/lpr* mice with thermal hypersensitivity had increased expression of TLR7 in the spinal dorsal horn. TLR7 was located in microglia in this region. Intrathecal administration of a TLR7 antagonist attenuated the thermal hypersensitivity in *MRL/lpr* mice, while administration of the TLR7 agonist induced thermal hypersensitivity in control mice. Pharmacological activation of spinal TLR7 in control mice recapitulated molecular, synaptic, and cellular changes in the spinal dorsal horn of *MRL/lpr* mice with thermal hyperalgesia. These alterations included activation of microglia and astrocytes, increased production of IL-1β and IL-18, upregulated expression of N-type voltage-gated calcium channels (Cav2.2), enhanced glutamatergic synaptic activity, and elevated neuronal activation. Our findings suggest that targeting TLR7 or downstream effectors may represent a promising strategy to alleviate chronic pain induced by SLE.

## 1. Introduction

Systemic lupus erythematosus (SLE) is a chronic autoimmune disease characterized by widespread inflammation and multi-organ involvement. Among its many manifestations, chronic pain is a debilitating symptom that affects a substantial proportion of patients, severely diminishing their quality of life [1,2,3]. Understanding the cellular and molecular mechanisms underlying SLE-induced chronic pain is therefore essential for developing novel therapeutic strategies to alleviate this condition.

The *MRL/lpr* lupus-prone mouse (Jackson Laboratory) is one of the most commonly used models of SLE [4,5,6,7], as it spontaneously develops key pathological features resembling human SLE. Similarly to SLE patients, *MRL/lpr* mice exhibit inflammation in multiple organs, including joints [8,9,10,11,12], kidneys [13], and other tissues [14,15,16]. Using *MRL/lpr* mice and their control strain (*MRL*), our previous studies have shown that *MRL/lpr* mice spontaneously develop hind paw hypersensitivity to radiant heat stimulation beginning at approximately 11 weeks of age, reaching a plateau between 14 and 16 weeks [17,18,19]. In lupus mice with chronic pain, primary nociceptive neurons in the dorsal root ganglia (L3–L6 segments) exhibit increased excitability, as indicated by elevated resting membrane potentials and reduced action potential thresholds and rheobases [20]. This neuronal hyperexcitability is accompanied by increased expression of proinflammatory cytokines, including TNFα and IL-1β, and reduced AMPK activity in the dorsal root ganglia [20]. In the lumbar spinal dorsal horn, lupus mice with chronic pain display pronounced glial activation and enhanced glutamatergic synaptic activity [17,21]. Elevated production of IL-1β and IL-18, together with increased p38 activity, contributes to the augmented glutamatergic transmission and chronic pain development in these mice [17,19]. However, the upstream signaling molecules that initiate these pathological changes remain poorly understood. Identifying such molecules could provide a foundation for developing novel analgesic strategies to treat lupus-induced chronic pain.

Toll-like receptor 7 (TLR7) plays a pivotal role in the pathogenesis of SLE [22,23,24]. Excessive activation of TLR7 by self-RNA–containing immune complexes is a key mechanism driving autoimmune responses in SLE [25,26]. TLR7 is a pattern recognition receptor localized to endosomal membranes, where it is activated by GU-rich single-stranded RNAs (ssRNAs) derived from viruses or endogenous sources such as damaged or apoptotic cells [27,28]. Activation of TLR7 triggers strong innate immune responses and production of proinflammatory cytokines [22,23]. TLR7 has been reported to be expressed in microglia in the forebrain [29,30], where aberrant activation of its signaling pathway contributes to neurodegenerative disorders such as Alzheimer’s [31,32,33] and Parkinson’s disease [34]. In the context of nociception, TLR7 is expressed in sensory neurons of the dorsal root ganglion (DRG), and its activation has been shown to contribute to neuropathic pain [35] and inflammatory pain induced by complete Freund’s adjuvant (CFA) [36]. However, whether and how TLR7 in the spinal dorsal horn contributes to the development of SLE-induced chronic pain remains unexplored.

N-type voltage-gated calcium channels (Cav2.2) belong to the high-voltage-activated calcium channel family and are primarily localized to primary nociceptive afferents terminating in the superficial laminae (I–II) of the spinal dorsal horn [37,38]. These channels mediate calcium influx into presynaptic terminals in response to membrane depolarization, triggering the release of excitatory neurotransmitters such as glutamate at the first synapse of the pain pathway [39,40]. Enhanced Cav2.2 activity has been implicated in the pathogenesis of pain induced by nerve injury [41,42,43], osteoarthritis [43], or formalin injection [44]. Currently, the role of Cav2.2 in the genesis of chronic pain induced by SLE remains unknown.

In the present study, we demonstrate that aberrant activation of TLR7 in the spinal dorsal horn contributes to enhanced neuronal activity and thermal hypersensitivity in *MRL/lpr* mice. Furthermore, we investigated the downstream signaling molecules involved in TLR7-mediated neuronal activation in the spinal dorsal horn.

## 2. Materials and Methods

### 2.1. Animals

Adult female *MRL/MpJ-fas^lpr^ (MRL/lpr)* and *MRL/MpJ* (*MRL* control) mice aged 8 to 16 weeks, procured from Jackson Laboratories (Bar Harbor, ME, USA), were used. Mice were housed four per cage in isolated rooms under a 12 h light–dark cycle. All experimental procedures were approved by the Institutional Animal Care and Use Committees of Mercer University and California Northstate University, and were conducted in full accordance with the National Institutes of Health guidelines for the care and use of laboratory animals. Female mice were used in this study because the incidence of SLE is approximately ninefold higher in females than in males [42]. Animals were randomly assigned to either the experimental or control group. Animals that died during the observation period were excluded from the analysis. The number of animals used per group was determined based on our previous studies [17,18,19].

### 2.2. Behavior Tests

Animals were positioned on a glass platform maintained at 30 °C and minimally confined in a Plexiglass chamber (12 × 20 × 15 cm), where they were given 1.5 h to habituate. Thermal sensitivity was assessed by applying a radiant heat source from beneath the glass to the mid-plantar surface of both left and right hind paws to elicit a withdrawal reflex. Paw withdrawal latency, defined as the time from stimulus onset to paw withdrawal, was measured. A 20 s cutoff was applied to prevent tissue injury. Each paw was tested three times with intervals of at least three minutes, and the mean withdrawal latency was calculated for each paw. All tests to study the behavioral pattern were performed by experimenters blinded to the mouse genotypes and treatment conditions.

### 2.3. Intrathecal Injection Procedure

Intrathecal (i.t.) administration of tested drugs to the spinal enlargement was performed through lumbar puncture as we and others described previously [45,46]. Mice were subjected to brief anesthesia using 2% isoflurane. Using 30-gauge needle, 5 μL of drug was intrathecally (i.t) injected through acute lumbar puncture between the L5 and L6 vertebrae. A successful penetration of the spinal compartment was indicated by a tail-flick.

### 2.4. Topical In Vivo Drug Application

Under urethane anesthesia (1.3–1.5 g/kg, i.p.), the L3–L6 spinal cord was revealed by laminectomy, and the spinal dura was carefully removed. Test drug(s) or vehicle (saline) was then administered onto the L3–L6 spinal region by placing a sterile cotton piece, soaked in the drug solution (35 °C), directly onto the exposed spinal cord for 2.5 h as we previously described [47,48]. Heart rate, respiration, and core body temperature were continuously monitored and maintained within physiological ranges. Immediately following to drug incubation, the dorsal portion of the L3–L6 spinal cord was dissected, immediately frozen in liquid nitrogen, and kept at −80 °C until Western blot experiments were conducted.

### 2.5. Western Blot Experiments

Animals were deeply anesthetized with urethane (1.3–1.5 g/kg, i.p.), after which the L3–L6 spinal segments were surgically exposed and harvested. The dorsal half of the spinal cord was isolated, quickly frozen in liquid nitrogen and stored at −80 °C for later use. Frozen tissues were thawed and homogenized in lysis buffer (50 mM Tris, pH 7.5, 150 mM NaCl, 1 mM EDTA, 0.1% SDS, 1% deoxycholic acid, 2 mM sodium orthovanadate, 100 mM NaF, 1% Triton X-100, 0.5 mM phenylmethylsulfonyl fluoride, 20 μM leupeptin, 100 IU mL^−1^ aprotinin) for about 30 min on ice with a hand-held homogenizer. Later, the homogenates were centrifuged for 20 min at 12,000× *g* at 4 °C, and the supernatants containing proteins were collected. Protein samples were quantified using the BCA method, and equal amounts (45 µg) were separated by electrophoresis on 10% SDS–polyacrylamide gels. Proteins were transferred to nitrocellulose membranes (Bio-Rad, Hercules, CA, USA), which were then blocked with 5% nonfat milk in TBST and incubated overnight at 4 °C with the following primary antibodies: rabbit anti-Cav2.2 (1:200; Cell Signaling Technology, Danvers, MA, USA), mouse anti-Iba1 (1:200; Millipore, Burlington, MA, USA), rabbit anti-GFAP (1:200; Cell Signaling Technology), rabbit anti-cathepsin B (1:200; Cell Signaling Technology), rabbit anti-IL-1β (1:200; Proteintech, Rosemont, IL, USA), rabbit anti-IL-18 (1:200; Bioss, Woburn, MA, USA), rabbit anti-MyD88 (1:200; Bioss), rabbit anti-phospho-ERK (1:200; Cell Signaling Technology), rabbit anti-phospho-p38 MAPK (1:100; Santa Cruz Biotechnology, Dallas, TX, USA), rabbit anti-c-Fos (1:200; Cell Signaling Technology), rabbit anti-TLR7 (1:200; Bioss), or rabbit anti-GAPDH (1:200; GeneTex, Irvine, CA, USA) as a loading control. The blots were then incubated for 2 h at room temperature with the appropriate secondary antibodies: IRDye^®^ 680LT goat anti-mouse IgG1 (1:5000; LI-COR Biosciences, Lincoln, Nebraska, USA) or IRDye^®^ 680RD donkey anti-rabbit IgG (1:5000; LI-COR Biosciences), diluted in TBST. Blots were subsequently scanned using the Odyssey^®^ CLx Imaging System (LI-COR Biosciences), and band intensities were quantified using ImageJ software (version 1.46; NIH, Bethesda, MD, USA). Protein levels were expressed as ratios relative to the loading control (GAPDH).

### 2.6. Immunohistochemical Experiments

Immunocytochemistry was employed to identify the specific cell types in the spinal dorsal horn that express TLR7. Animals were deeply anesthetized with urethane (1.3–1.5 g/kg, i.p.) and transcardially perfused with heparinized phosphate-buffered saline (PBS, pH 7.35), followed by fixation with 4% formaldehyde in 0.1 M PBS (pH 7.35). The L3–L6 spinal cord segments were dissected and post-fixed in the same fixative for 24 h at 4 °C. The tissues were then cryoprotected by 30% sucrose in 0.1 M PBS at 4 °C. Transverse sections (30 µm) were cut on a freezing microtome at −20 °C, processed as free-floating sections, and collected in 0.1 M PBS. Before staining, sections were washed three times with PBS and incubated for 1 h at room temperature in a blocking solution containing 10% normal goat serum and 0.3% Triton X-100 in 0.1 M PBS (pH 7.35). Sections were incubated at room temperature for 1 h, followed by overnight incubation at 4 °C with the following primary antibodies: rabbit anti-TLR7 (1:200; Bioss, Woburn, MA, USA), goat anti-Iba1 (microglial marker, 1:100; Novus, Centennial, CO, USA), mouse anti-GFAP (astrocytic marker, 1:500; Cell Signaling Technology, Danvers, MA, USA), and mouse anti-NeuN (neuronal marker, 1:500; Cell Signaling Technology, Danvers, MA, USA). After three washes in 0.1 M PBS, sections were incubated for 2 h at room temperature with the appropriate secondary antibodies, including Texas Red (1:500; Vector Laboratories, Newark, CA, USA) and Alexa Fluor 488 (1:500; Life Technologies, Gaithersburg, MD, USA). Finally, the sections were rinsed thrice in PBS and mounted onto gelatin-coated slides. They were allowed to air-dry, and then overlaid with Vectashield mounting medium (Vector Laboratories). Immunofluorescence was visualized and documented using a Zeiss Apotome microscope (Zeiss, Oberkochen, Germany).

### 2.7. Spinal Slice Preparations for Whole-Cell Patch Clamp Recording

Experiments were conducted using sixteen-week-old mice. Transverse spinal cord slices (350 µm thick) from the L3–L6 region were prepared following our previously described methods [49,50]. In brief, mice were profoundly anesthetized with 2.5–3% isoflurane, the lumbar enlargement of the spinal cord was surgically exposed and excised. The dissected lumbar segment was immediately transferred into ice-cold sucrose-based artificial cerebrospinal fluid (aCSF, 300 mL) equilibrated with 95% O_2_ and 5% CO_2_, and then thoroughly rinsed to remove residual blood. The sucrose aCSF contained 234 mM sucrose, 3.6 mM KCl, 1.2 mM MgCl_2_, 2.5 mM CaCl_2_, 1.2 mM NaH_2_PO_4_, 12.0 mM glucose, and 25.0 mM NaHCO_3_. Following the removal of the pia-arachnoid membrane, the L3-6 spinal segment—verified by its prominent dorsal roots—was secured to a cutting support using cyanoacrylate adhesive. The support was then mounted onto the stage of a vibratome (Series 1000, Technical Products International, St. Louis, MO, USA) for slicing. Transverse sections of the spinal cord (350 μm thick) were cut within the chilled sucrose aCSF environment before being moved to oxygenated Krebs solution for pre-incubation at 35 °C. The Krebs solution used in this process contained the following concentrations (mM): 117.0 NaCl, 3.6 KCl, 1.2 MgCl_2_, 2.5 CaCl_2_, 1.2 NaH_2_PO_4_, 11.0 glucose, and 25.0 NaHCO_3_ at a constant temperature of 35 °C.

### 2.8. Whole-Cell Patch Clamp Recording and Analysis of Miniature Excitatory Postsynaptic Currents (mEPSCs) from Neurons in the Spinal Dorsal Horn

Whole-cell patch-clamp recordings were conducted using methods established in earlier studies [51,52]. Following the pre-incubation period, individual spinal cord slices were transferred to a 1.5 mL recording chamber, where they were continuously perfused with Krebs solution, which was maintained at 35 °C and constantly equilibrated with 95% O_2_ and 5% CO_2_. Recording pipettes were crafted from borosilicate glass to a resistance of 3–5 MΩ and filled with an internal solution comprising (in mM): 135 potassium gluconate, 5 KCl, 2 MgCl_2_, 0.5 CaCl_2_, 5 HEPES, 5 EGTA, 5 Mg-ATP, 0.5 Na-GTP, and 10 QX-314. Neurons located in lamina I and outer lamina II (IIo) of the dorsal horn were visualized under a microscope, and whole-cell configurations were achieved using a three-dimensional motorized manipulator (Sutter Instrument, Novato, CA, USA). After the electrode made contact with the cell, gentle negative pressure was applied to break into the cell membrane [49,50,53].

Miniature excitatory postsynaptic currents (mEPSCs) were recorded from neurons that received monosynaptic inputs from primary afferents, based on previously established criteria [52,53]. To isolate these specific events, the bath solution included 1 μM tetrodotoxin (TTX) to block voltage-gated sodium channels, 10 μM bicuculline to block GABA_A_ receptors, and 5 μM strychnine to block glycine receptors. Cells were voltage-clamped at −60 mV throughout the recording sessions. Signals were acquired with an Axopatch 700B amplifier, digitized at 10 kHz, and subjected to offline analysis. Access resistance was continuously monitored, maintained within 10–20 MΩ, and any experiment where this resistance varied by more than 20% was stopped. The frequency and amplitude of mEPSCs were quantified using the MiniAnalysis peak detection program (Synaptosoft Inc., Decatur, GA, USA) and averaged over 2 min intervals before, during, and after the application of drugs, which were applied via bath perfusion.

### 2.9. Materials

Bicuculline, strychnine, DSR 6434, and tetrodotoxin were obtained from Tocris (Bristol, UK). ODN 2088 was purchased from Miltenyi BiotecI (Auburn, CA, USA). All other reagents were obtained from Sigma-Aldrich (St. Louis, MO, USA).

### 2.10. Data Analysis

Data are expressed as mean ± standard error (SEM). Behavioral data across time points within the same group were examined using one-way repeated-measures ANOVA, while two-way repeated-measures ANOVAs were applied to compare differences between groups across multiple time points. When significant effects were detected, Tukey’s post hoc tests were performed to identify specific group or time point differences. For comparisons between two datasets, Student’s paired *t*-tests were used for within-group comparisons and unpaired *t*-test for between-group comparisons. Statistical significance was defined as *p* < 0.05. All analyses were conducted using GraphPad Prism 10 (GraphPad Software Inc., Boston, MA, USA).

## 3. Results

### 3.1. MRL/lpr Mice Spontaneously Exhibited Thermal Hypersensitivity Accompanied by Increased Neuronal Activation in the Spinal Dorsal Horn

**Figure 1:**
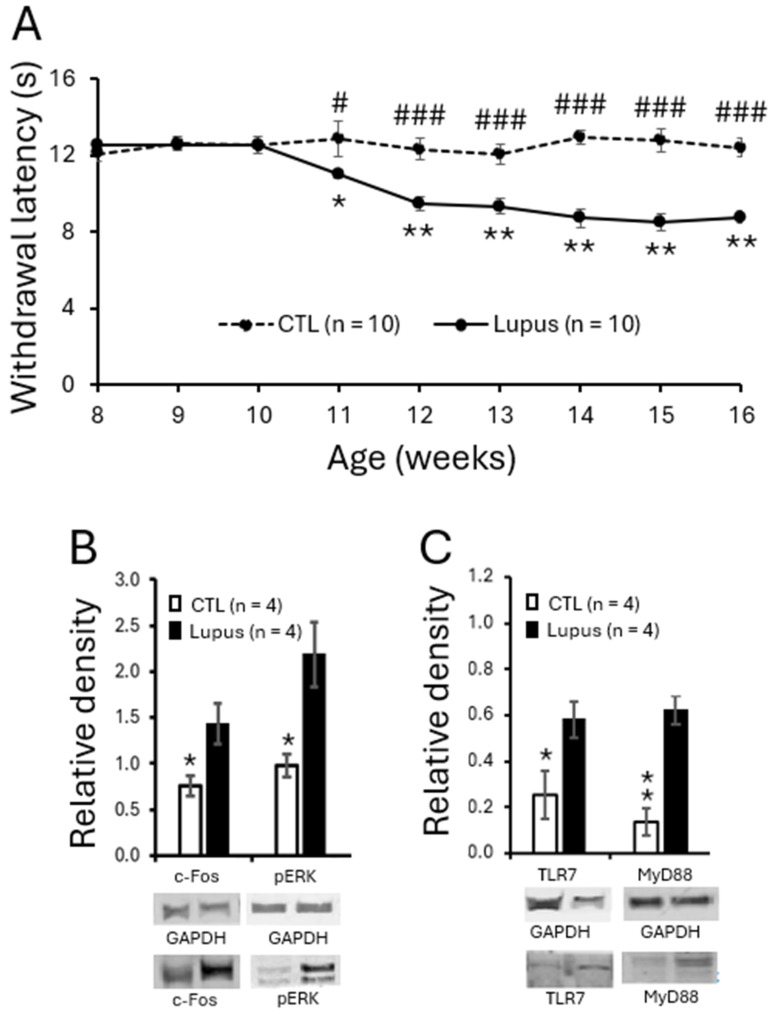
*MRL/lpr* mice spontaneously exhibited thermal hypersensitivity, accompanied by increased neuronal activation and elevated expression of TLR7 and MyD88 in the spinal dorsal horn. (**A**) Line plots show measurements (mean ± SEM) of withdrawal response latencies of the hind paw to radiant heat stimulation from ages of 8 to 16 weeks in *MRL* control (*n* = 10) and *MRL/lpr* (*n* = 10) mice. Comparisons between the age of 8 weeks to each following week in *MRL/lpr* mice are indicated with * while comparisons between *MRL* control and *MRL/lpr* mice at each time point are labeled with #. (**B**) Bar graphs show mean (±SEM) ratios of c-Fos and phosphorylated ERK (pERK) to GAPDH protein expression in the spinal dorsal horn of *MRL* control (*n* = 4) and *MRL/lpr* (*n* = 4) mice. (**C**) Bar graphs show mean (±SEM) ratios of TLR7 and MyD88 to GAPDH protein expression in the spinal dorsal horn of *MRL* control (*n* = 4) and *MRL/lpr* (*n* = 4) mice. Samples of protein expression for molecules in each group are shown below. *, # *p* < 0.05; ** *p* < 0.01; ### *p* < 0.001.

To assess the development of chronic pain in MRL/lpr mice, hind paw sensitivity to radiant heat was measured weekly from 8 to 16 weeks of age in female MRL/lpr and control mice. The withdrawal latencies to radiant thermal stimulation were comparable and remained stable in both groups between 8 and 10 weeks of age. However, beginning at 11 weeks, *MRL/lpr* mice exhibited a significant reduction in withdrawal latency (*n* = 10, *p* < 0.05), which progressively declined and reached a plateau between 14 and 16 weeks. Specifically, withdrawal latencies in *MRL/lpr* mice decreased from 12.51 ± 0.14 s at 8 weeks to 11.04 ± 0.21 s at 11 weeks (*p* < 0.05), and further to 8.78 ± 0.29 s at 16 weeks (*p* < 0.01). In contrast, *MRL* control mice (*n* = 10) showed no significant changes in withdrawal latency during the same period (Figure 1A). Consequently, withdrawal latencies in *MRL/lpr* mice from 11 to 16 weeks were significantly shorter than those in *MRL* control mice. These findings are consistent with our previous reports [17,18,19] and indicate that *MRL/lpr* mice begin to develop thermal hyperalgesia at 11 weeks of age, which persists through 16 weeks. To investigate the mechanisms underlying the thermal hyperalgesia in lupus mice, all subsequent experiments in this study were performed using 16-week-old *MRL/lpr* mice with thermal hypersensitivity and age-matched *MRL* control mice.

To determine whether excessive neuronal activation in the spinal dorsal horn is associated with the pain phenotype in *MRL/lpr* mice, we examined the protein expression of two well-established markers for neuronal activity—c-Fos and phosphorylated ERK (p-ERK) [54,55,56]—using Western blot analysis. As shown in Figure 1B, the protein levels of c-Fos and p-ERK in the spinal dorsal horn of 16-week-old *MRL/lpr* mice (*n* = 4) with thermal hypersensitivity were significantly increased compared to those in age-matched *MRL* controls (*n* = 4), indicating enhanced neuronal activation in the spinal dorsal horn of *MRL/lpr* mice.

### 3.2. TLR7 Protein Expression Was Elevated in Spinal Dorsal Horn and Present in Microglia

Given the crucial role of TLR7 in the development of peripheral symptoms in SLE and the limited understanding of its role in the CNS, we investigated whether spinal TLR7 signaling contributes to the genesis of chronic pain associated with SLE. We found that TLR7 protein expression in the spinal dorsal horn was increased in *MRL/lpr* mice at 16 weeks of age (*n* = 4) compared with age-matched *MRL* control mice (*n* = 4) (Figure 1C). MyD88 is a downstream adaptor molecule for all the TLRs except TLR3 and IL-1β receptor family [57,58] and its expression is increased upon activation of TLRs [59,60]. We next examined whether its expression is also increased in lupus mice. As expected, MyD88 protein expression was significantly enhanced in lupus mice (Figure 1C). The increased MyD88 is consistent with TLR7 activation in SLE-associated pain although our data do not exclude potential contributions from other TLRs.

**Figure 2:**
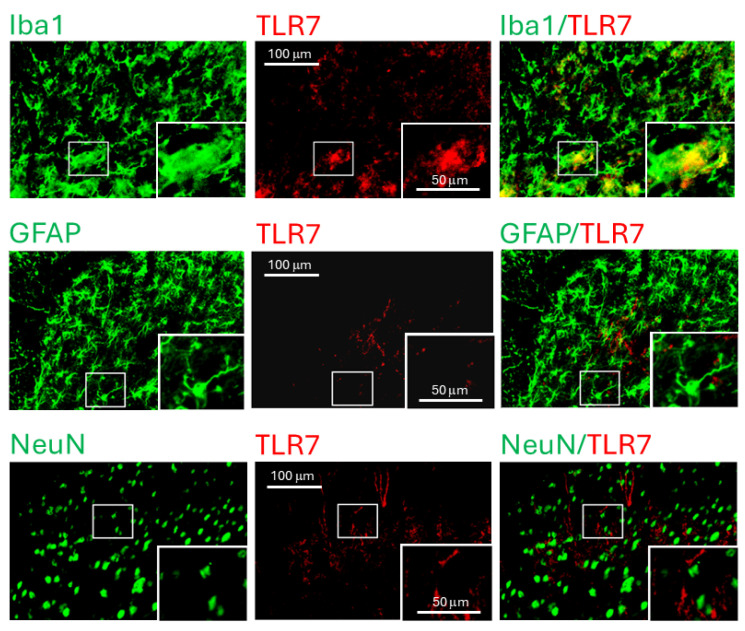
TLR7 protein was detected in microglia within the spinal dorsal horn of *MRL/lpr* mice. Fluorescent images were captured from the dorsal region of the spinal dorsal horn in 16-week-old *MRL/lpr* mice, with the inset showing an enlarged view of the area indicated by the rectangular box. In the spinal slices, microglia, astrocytes, and neurons were labeled green using Iba1, GFAP, and NeuN antibodies, respectively, while TLR7 was labeled in red. Colocalization of TLR7 with the respective cell markers is shown on the right.

To identify the cellular localization of TLR7 in the spinal dorsal horn, we performed immunohistochemical staining on spinal sections from L3–L6 segments of *MRL/lpr* mice with thermal hyperalgesia and age-matched *MRL* controls. TLR7 immunoreactivity (red) was co-localized exclusively with the microglial marker Iba1 (green), but not with the astrocytic marker GFAP (green) or the neuronal marker NeuN (green), in both *MRL/lpr* mice (Figure 2) and control mice (Supplementary Figure S1). These findings indicate that TLR7 is specifically expressed in microglia in the spinal dorsal horn. The increased TLR7 expression observed in *MRL/lpr* mice with thermal hyperalgesia suggests that enhanced microglial TLR7 activity may cause chronic pain associated with SLE.

### 3.3. Intrathecal Administration of TLR7 Antagonists Reversed Thermal Hyperalgesia in MRL/lpr Mice

**Figure 3:**
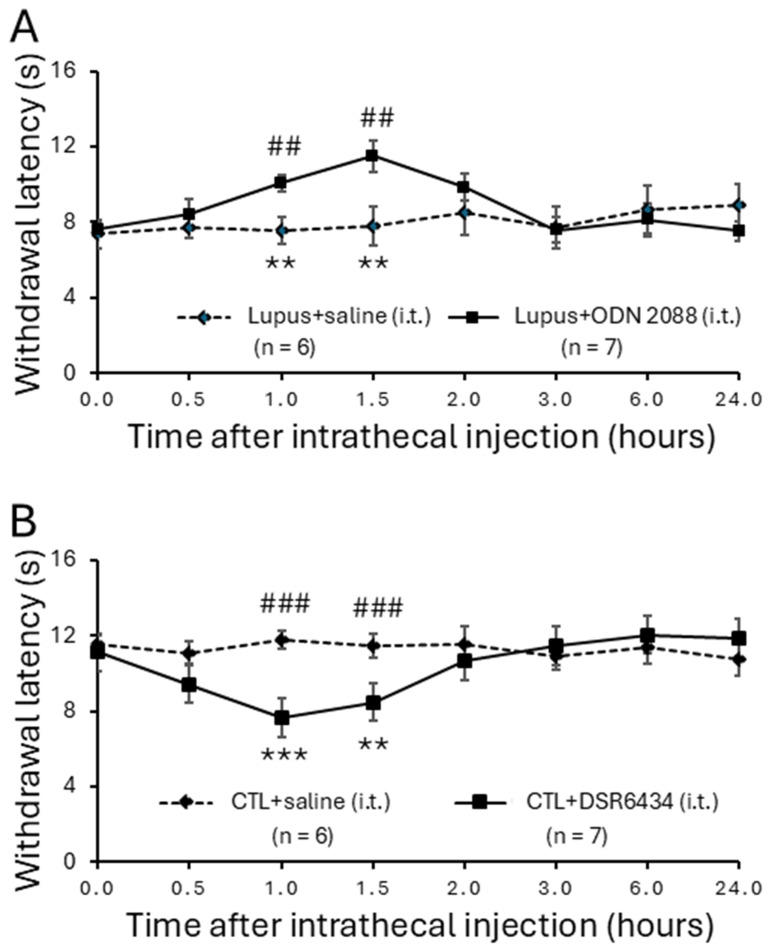
Intrathecal administration of TLR7 antagonists reversed thermal hyperalgesia in *MRL/lpr* mice while injection with TLR7 agonists induced thermal hyperalgesia in control mice. (**A**) Line plots show hind paw withdrawal latencies to radiant heat stimuli (mean ± SEM) in *MRL/lpr* mice measured at baseline (0) and 0.5, 1.0, 1.5, 2.0, 3.0, 6.0, and 24.0 h following intrathecal TLR7 antagonist (ODN2088; concentration: 10 μg/mL; volume: 5 μL) or vehicle saline solution (5 μL) administration. Comparisons between the baseline to each time point after the ODN2088 injection in the same group are indicated with * while comparisons between *MRL/lpr* mice treated with ODN2088 and *MRL/lpr* mice treated with saline at each time point are labeled with #. (**B**) Line plots show hind paw withdrawal latencies (mean ± SEM) in *MRL* control mice measured at baseline (0) and 0.5, 1.0, 1.5, 2.0, 3.0, 6.0, and 24.0 h following intrathecal TLR7 agonist (DSR 6434; concentration: 20 μMol; volume: 5 μL) or vehicle saline solution (5 μL) administration. Comparisons between the baseline to each time point after the DSR 6434 injection in the same group are indicated with * while comparisons between *MRL* control mice treated with DSR 6434 and *MRL* control mice treated with saline at each time point are labeled with #. **, ## *p* < 0.01; ***, ### *p* < 0.001.

To investigate the causal role of TLR7 in lupus-associated pain, we examined whether intrathecal administration of the TLR7 blocker ODN 2088 attenuates hind paw hypersensitivity to radiant heat in *MRL/lpr* mice. Sixteen-week-old *MRL/lpr* mice with established thermal hyperalgesia were randomly assigned to an ODN 2088-treated group or a vehicle (saline)-treated group. After baseline withdrawal latencies were recorded, ODN 2088 (10 μg/mL, 5 μL) was administered intrathecally via lumbar puncture [35,36]. Withdrawal latencies in *MRL/lpr* mice increased significantly, from 7.61 ± 0.34 s (*n* = 7) before injection to 10.06 ± 0.43 s (*n* = 7) at 60 min post-injection. The analgesic effect peaked at 90 min and diminished by 2 h after injection (Figure 3A). In contrast, *MRL/lpr* mice receiving intrathecal vehicle (saline) injections (*n* = 6) showed no significant change in withdrawal latency. Comparison between vehicle-treated (*n* = 6) and ODN 2088-treated (*n* = 7) *MRL/lpr* mice confirmed that ODN 2088 significantly increased withdrawal latencies at 60 min and 90 min post-injection (*p* < 0.01), indicating that TLR7 blockade alleviates thermal hyperalgesia in lupus mice.

### 3.4. Intrathecal Administration of TLR7 Agonists Induced Thermal Hyperalgesia in Control Mice

Next, we investigated whether activation of spinal TLR7 by a TLR7 agonist (DSR 6434) could induce thermal hyperalgesia in *MRL* control mice. Sixteen-week-old *MRL* control mice were assigned to receive intrathecal injections of either DSR 6434 or vehicle (saline). Intrathecal administration of DSR 6434 (20 μM, 5 μL) significantly reduced the withdrawal latency to radiant heat stimulation from 11.15 ± 0.49 s (*n* = 7) pre-injection to 7.66 ± 0.48 s at 60 min (*n* = 7; *p* < 0.001) and 8.46 ± 0.61 s at 90 min post-injection (*n* = 7; *p* < 0.01) (Figure 3B). This effect disappeared by 2 h after injection. In contrast, *MRL* control mice (*n* = 6) receiving intrathecal vehicle injections (5 μL saline, i.t.) showed no significant change in withdrawal latency during the same observation period (Figure 3B). Therefore, the withdrawal latencies of DSR 6434–treated mice were significantly shorter than those of vehicle-treated controls at 60 min and 90 min post-injection (*p* < 0.001). Along with the findings in Figure 3A, these results demonstrate that activation of spinal TLR7 contributes to the genesis of thermal hyperalgesia in *MRL/lpr* mice.

### 3.5. Activation of Spinal TLR7 with the Exogenous TLR7 Agonist Increased TLR7 and MyD88 Expression and Induced Spinal Neuronal Activation

**Figure 4:**
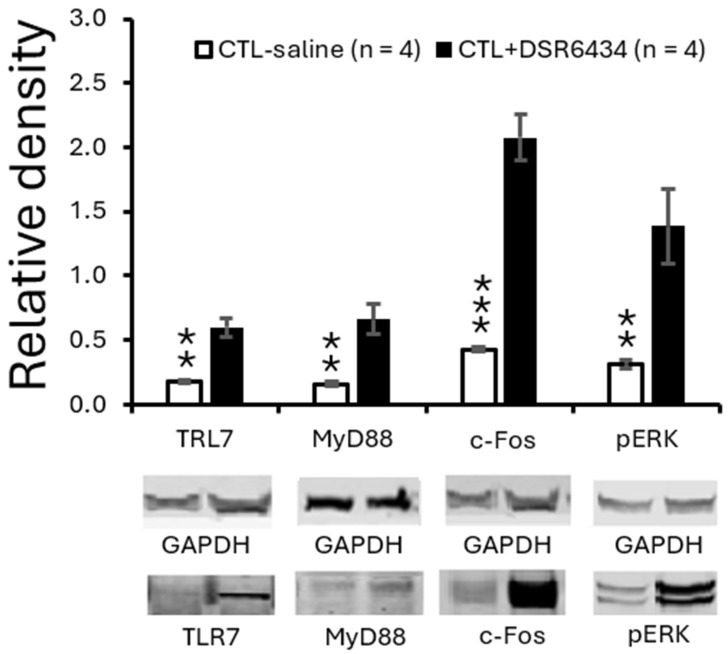
Activation of spinal TLR7 with an exogenous agonist increased TLR7 and MyD88 expression and induced spinal neuronal activation. Bar graphs show mean (±SEM) ratios of TLR7, MyD88, c-Fos, and pERK to GAPDH in the spinal dorsal horn of *MRL* control mice treated with DSR 6434 (DSR 6434 (2 μM) (*n* = 4), and those with saline (*n* = 4). Samples of protein expression for molecules in each group are shown below. ** *p* < 0.01, *** *p* < 0.001.

Given that *MRL/lpr* mice with thermal hyperalgesia exhibit increased TLR7 protein expression, we next investigated whether this increase reflects enhanced TLR7 activation. To activate TLR7 in the spinal dorsal horn, a piece of cotton soaked with DSR 6434 (2 μM in saline, 35 °C) was placed on the dorsal surface of the L3–L6 spinal segments for 2.5 h in *MRL* control mice anesthetized with urethane (1.3–1.5 g/kg, i.p.). *MRL* control mice treated with saline in the same manner served as controls. Activation of TLR7 with DSR 6434 significantly increased protein expression of TLR7 and its downstream signaling molecule MyD88 in the spinal dorsal horn (*n* = 4, *p* < 0.01) compared with saline-treated control mice (Figure 4). Concurrently, protein levels of c-Fos and p-ERK were also elevated relative to those in saline-treated controls (Figure 4). These results demonstrate that TLR7 protein expression in the spinal dorsal horn is upregulated by TLR7 activation and suggest that endogenous activation of TLR7 in *MRL/lpr* mice with thermal hyperalgesia accounts for the enhanced TLR7 protein expression. The concomitant increases in c-Fos and p-ERK further indicate that activation of spinal TLR7 contributes to heightened neuronal activity in the spinal dorsal horn of *MRL/lpr* mice.

### 3.6. Activation of Spinal TLR7 in MRL Control Mice Enhanced Activation of Microglia and Astrocytes, P38 MAPK (P38) Phosphorylation and Production of IL-1β and IL-18

**Figure 5:**
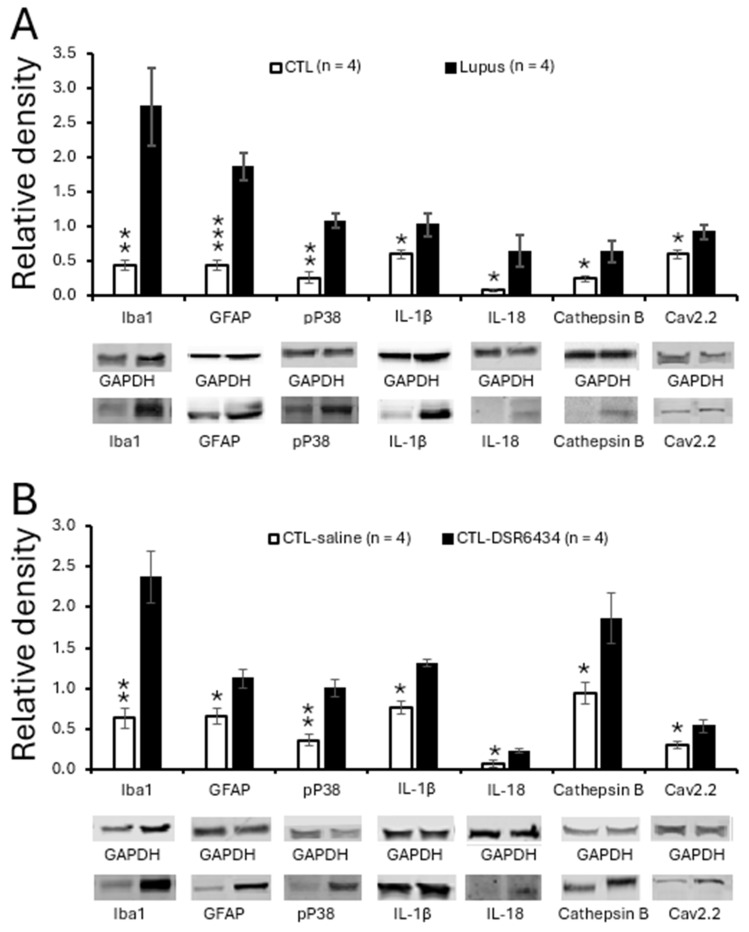
Spinal TLR7 activation in control mice recapitulated signaling molecular changes in lupus mice with chronic pain. (**A**) Bar graphs show the mean (±SEM) ratios of Iba1, GFAP, pP38, IL-1β, IL-18, cathepsin B, and Cav2.2 to GAPDH in the spinal dorsal horn of *MRL* control (*n* = 4) and MRL/lpr (*n* = 4) mice. (**B**) Bar graphs show mean (±SEM) ratios of Iba1, GFAP, pP38, IL-1β, IL-18, cathepsin B, and Cav2.2 to GAPDH in the spinal dorsal horn of *MRL* control mice (*n* = 4) treated with a TLR7 agonist, DSR 6434 (DSR 6434 (2 μM), and those with saline (*n* = 4). Samples of protein expression for molecules in each group are shown below. * *p* < 0.05, ** *p* < 0.01, *** *p* < 0.001.

Concurrent with the enhanced TLR7 protein expression, *MRL/lpr* mice with chronic pain exhibited increased activation of microglia and astrocytes, as evidenced by elevated protein levels of Iba1 (a microglial marker) and GFAP (an astrocytic marker) in the spinal dorsal horn. These changes were accompanied by increased phosphorylation of P38 MAPK (pP38) and elevated levels of the proinflammatory cytokines IL-1β and IL-18. In the same region, expression of cathepsin B—a protease that converts pro-IL-1β and pro-IL-18 into their mature forms [61,62]—was also increased (Figure 5A). Both IL-1β [63,64,65] and IL-18 [66] are known to be synthesized and released by microglia [67,68], and we previously demonstrated that IL-1β released from activated microglia can induce astrocyte activation [41]. Since TLR7 is expressed in microglia (Figure 2), we next investigated whether exogenous activation of TLR7 with the agonist DSR 6434 induces microglial and astrocyte activation, as well as changes in pP38, IL-1β, IL-18, and cathepsin B protein levels. Spinal cords of *MRL* control mice were incubated with DSR 6434 (2 μM) or saline for 2.5 h. We found that DSR 6434 treatment significantly increased protein levels of Iba1, GFAP, pP38, IL-1β, IL-18, and cathepsin B in the spinal dorsal horn (*n* = 4) compared with saline-treated controls (*n* = 4, Figure 5B). These results indicate that TLR7 activation in the spinal cord promotes microglial and astrocytic activation as well as the production of IL-1β and IL-18, recapitulating the signaling changes observed in lupus mice with chronic pain.

### 3.7. Activation of Spinal TLR7 in MRL Control Mice Enhanced Protein Expression of N-Type Voltage-Gated Calcium Channels (Cav2.2)

To determine whether Cav2.2 protein expression is altered in lupus mice with chronic pain, we compared Cav2.2 levels in the spinal dorsal horn between *MRL/lpr* mice and age-matched control mice. We found that Cav2.2 expression was significantly higher in lupus mice with chronic pain (*n* = 4) than in control mice (*n* = 4; *p* < 0.05; Figure 5A). Furthermore, activation of spinal TLR7 by incubating normal spinal cords (*n* = 4) with DSR 6434 (2 μM) for 2.5 h significantly increased Cav2.2 expression in the dorsal horn (Figure 5B), reproducing the pathological change observed in lupus mice.

### 3.8. Activation of TLR7 in the Spinal Dorsal Horn in Lupus Mice Enhanced Presynaptic Glutamate Release and Postsynaptic AMPA Glutamate Receptor Activity in Superficial Dorsal Horn Neurons

**Figure 6:**
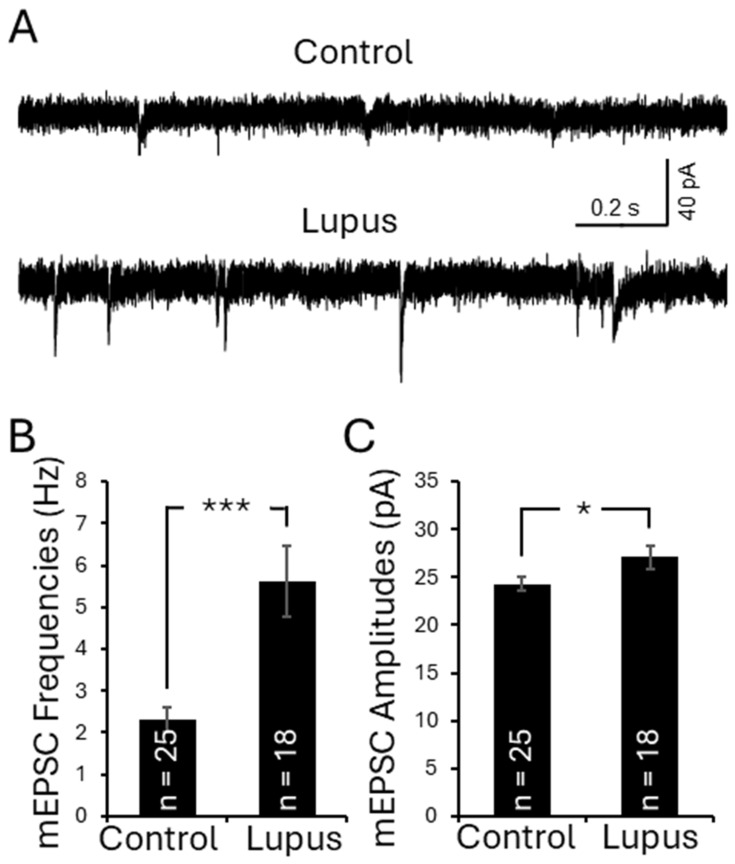
Lupus mice had increases in presynaptic glutamate release and postsynaptic AMPA receptor activity in the superficial dorsal horn neurons. (**A**) Samples of mEPSC recordings from normal mice and lupus mice are shown. Bar graphs show comparisons of mean (±SEM) mEPSC frequencies (**B**) and amplitudes (**C**) between lupus mice (*n* = 9) and controls (*n* = 13). The number of neurons analyzed for each group is indicated on each bar. * *p* < 0.05, *** *p* < 0.01.

Finally, we investigated the synaptic mechanisms underlying the enhanced neuronal activity induced by TLR7 activation. Glutamatergic synaptic activity is a key determinant of neuronal activation in the spinal dorsal horn [69,70]. To examine how TLR7 activation affects synaptic function, we performed whole-cell voltage-clamp recordings of AMPA receptor-mediated miniature excitatory postsynaptic currents (mEPSCs) from neurons in the superficial dorsal horn (laminae I and II outer layer, IIo) [51,71]. Neurons in this region are predominantly excitatory and receive direct inputs from nociceptive primary afferents, making them a critical hub for pain signal processing [69,70]. We first compared mEPSC frequency and amplitude between lupus mice (*n* = 9) and control mice (*n* = 13). As shown in Figure 6, both mEPSC frequency (5.62 ± 0.85 Hz; *n* = 18) and amplitude (27.07 ± 1.46 pA; *n* = 18) in lupus mice were significantly increased in comparison with the mEPSC frequency (2.31 ± 0.29 Hz; *n* = 25) and amplitude (24.30 ± 0.75 pA; *n* = 25) observed in controls. These findings indicate enhanced presynaptic glutamate release as well as increased postsynaptic glutamate receptor responsiveness in lupus mice.

**Figure 7:**
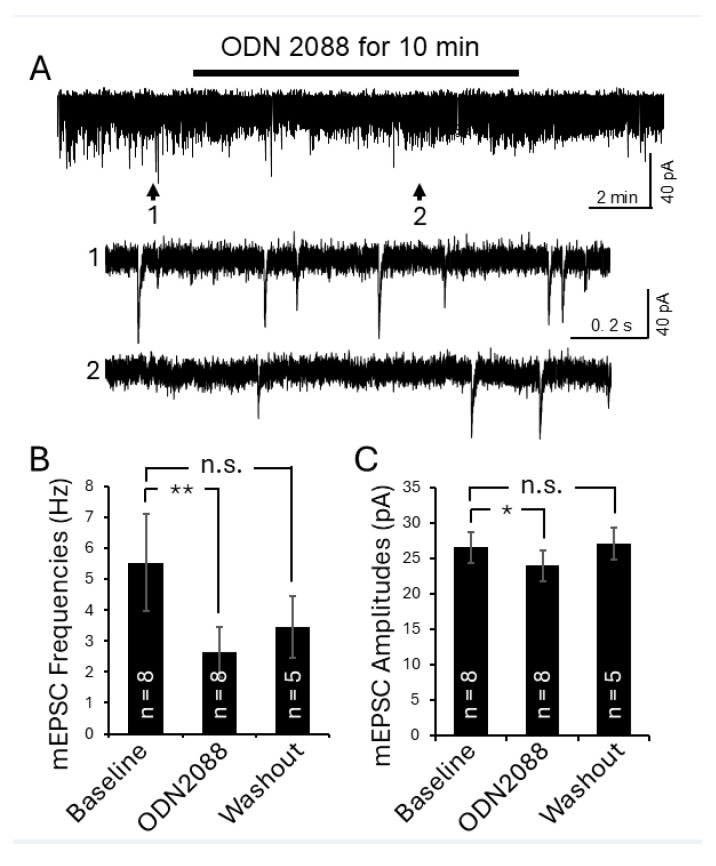
Inhibition of TLR7 in the spinal dorsal horn of lupus mice reduced presynaptic glutamate release and postsynaptic AMPA glutamate receptor activity in the superficial dorsal horn neurons. (**A**) Raw data show the effect of TLR7 inhibition by the TLR7 inhibitor ODN 2088 on mEPSCs recorded from a superficial dorsal horn neuron in a lupus mouse. Bath perfusion of ODN 2088 (bath concentration: 100 ng/mL) reversibly and significantly attenuated mEPSC frequency and amplitude. Arrows indicate the time points of the recordings that are shown below at an expanded time scale. Bar graphs show mean (±SEM) mEPSC frequencies (**B**) and amplitudes (**C**) from lupus mice before, during, and after ODN 2088 bath-perfusion. The number of neurons analyzed for each group is indicated on each bar. These data were collected from 4 lupus mice. * *p* < 0.05, ** *p* < 0.01; n.s., no significance.

We next examined whether blocking TLR7 could attenuate these pathological changes in lupus mice. After recording baseline mEPSC activity, the TLR7 inhibitor (ODN 2088; bath concentration: 100 ng/mL) was applied for 10 min. As shown in Figure 7, bath perfusion of ODN 2088 significantly reduced mEPSC frequency from 5.53 ± 1.58 Hz (*n* = 8) to 2.65 ± 0.82 Hz (*n* = 8) and decreased mEPSC amplitude from 26.60 ± 2.17 pA (*n* = 8) to 23.87 ± 2.18 pA (*n* = 8) in lupus mice (*n* = 4). These effects were reversed upon washout of ODN 2088. These results indicate that increased activation of TLR7 in the spinal dorsal horn is responsible for the increase in presynaptic glutamate release and postsynaptic AMPA receptor activity in lupus mice.

**Figure 8:**
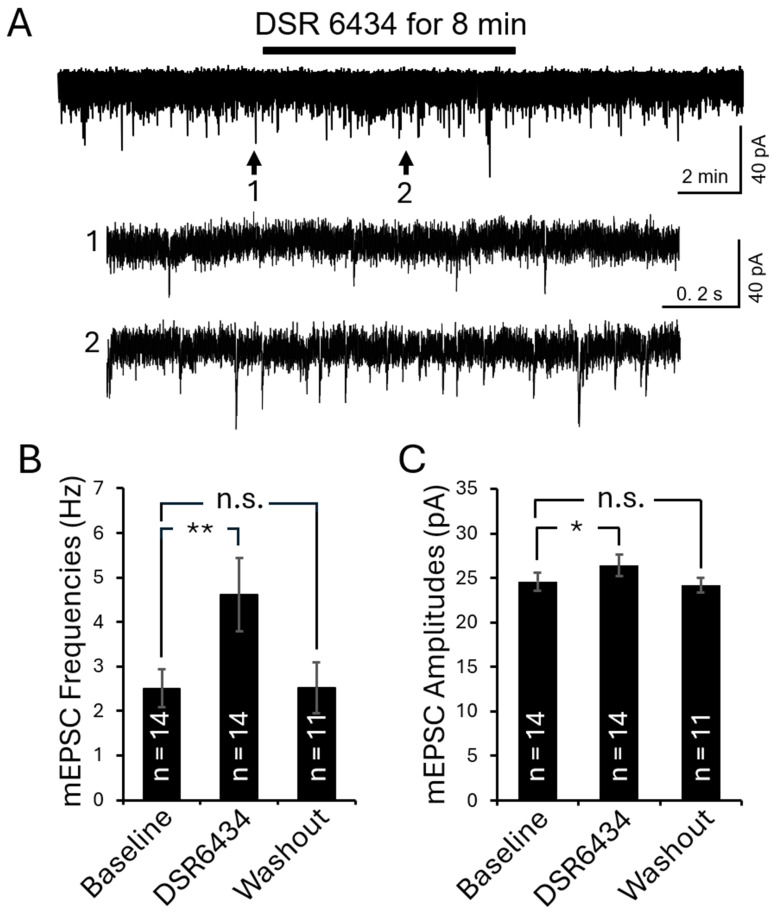
Activation of TLR7 in the spinal dorsal horn of control mice enhanced presynaptic glutamate release and postsynaptic AMPA glutamate receptor activity in the superficial dorsal horn neurons. (**A**) Raw data showing the effect of TLR7 activation by the TLR7 agonist DSR 6434 on mEPSCs recorded from a superficial dorsal horn neuron in an *MRL* control mouse. Bath perfusion of DSR 6434 (final concentration in the recording chamber: 200 nM) reversibly and significantly increased mEPSC frequency and amplitude. Arrows indicate the time points of the recordings that are shown below at an expanded time scale. Bar graphs show mean (±SEM) mEPSC frequencies (**B**) and amplitudes (**C**) from *MRL* control mice before, during, and after DSR 6434 bath-perfusion. The number of neurons analyzed for each group is indicated on each bar. These data were collected from 8 mice. * *p* < 0.05, ** *p* < 0.01; n.s., no significance.

Lastly, we examined the effects of TLR7 activation on mEPSCs in 16-week-old *MRL* control mice (*n* = 8) using the TLR7 agonist DSR 6434. After recording baseline mEPSC activity, DSR 6434 (concentration in the recording chamber: 200 nM) was applied to the recording chamber for 8 min. Activation of TLR7 significantly increased mEPSC frequency from 2.50 ± 0.42 Hz (*n* = 14 neurons) to 4.61 ± 0.82 Hz (*n* = 14 neurons; *p* < 0.01), and mEPSC amplitude from 24.58 ± 1.04 pA (*n* = 14 neurons) to 26.39 ± 1.23 pA (*n* = 14 neurons; *p* < 0.05) (Figure 8). These effects were reversed upon washout of DSR 6434 (Figure 8). These results demonstrate that TLR7 activation in the spinal dorsal horn facilitates presynaptic glutamate release and enhances postsynaptic AMPA receptor responses in superficial dorsal horn neurons.

## 4. Discussion

In this study, we elucidated the role of spinal TLR7 in the pathogenesis of chronic pain induced by SLE and its underlying mechanisms. Notably, we demonstrated for the first time that TLR7 is expressed in microglia in the spinal dorsal horn, and that *MRL/lpr* mice with thermal hyperalgesia exhibit enhanced TLR7 activation in this region. TLR7 activation induces microglial and astrocytic activation and promotes IL-1β and IL-18 production of in the spinal dorsal horn. Furthermore, TLR7 activation increases Cav2.2 protein expression and enhances glutamatergic synaptic activity in dorsal horn neurons. These findings advance our understanding of the role of TLR7 in the SLE pathogenesis, and the cellular and molecular mechanisms underlying SLE-induced central sensitization in the spinal cord. Our study provides a foundation for developing novel and more effective therapeutic strategies to alleviate chronic pain in patients with SLE.

### 4.1. Role of TLR7 in the SLE Pathogenesis

SLE is an autoimmune disease in which impaired clearance of apoptotic cells leads to accumulation of cellular debris, which continuously exposes the immune system to self-derived nuclear antigens. Nucleic acids from this debris engage TLR7, triggering innate immune activation in plasmacytoid dendritic cells, myeloid cells, and B lymphocytes [72,73]. While the role of TLR7 in peripheral SLE pathology is well established [74], its involvement in SLE-induced CNS disorders remains poorly understood. For example, while one study reported increased TLR7 expression in the brains of NZB/W F1 mice, a model of SLE [75], another study found that TLR7 deletion ameliorated peripheral symptoms but did not improve neuropsychiatric manifestations of SLE [76]. Our study underscores the role of TLR7 in SLE-induced neuropathology by demonstrating that spinal TLR7 signaling is critically involved in the genesis of SLE-induced chronic pain. Furthermore, the factors regulating TLR7 signaling remain incompletely understood. Human studies have shown that specific polymorphisms result in increased TLR7 expression [77]. In the present study, we found that TLR7 protein expression is upregulated following activation by the TLR7 agonist DSR 6434, suggesting that increased exposure to endogenous TLR7 activators may contribute to the enhanced TLR7 signaling in SLE pathogenesis [78,79].

### 4.2. Role of TLR7 in Pain

Studies on the role of TLR7 in pain signaling have primarily focused on peripheral sensory neurons. TLR7 is expressed in small-diameter sensory neurons of the DRG [80], and its expression is increased in DRG neurons ipsilateral to nerve injury [35,81]. TLR7 mRNA levels are elevated in the medulla of rats with chronic constriction injury of the infraorbital nerve [82]. In the spinal dorsal horn, expression of the TLR7 activator miRNA lethal-7 (let-7) is increased in mice with CFA-induced inflammatory pain [36]. Intrathecal injection of a let-7b antagomir inhibits inflammatory pain and spinal synaptic plasticity induced by CFA, effects attributed to the action of let-7b on primary afferent terminals and microglial activation in the dorsal horn [36]. Intra-articular injection of a TLR7 antagonist also attenuates mechanical allodynia in a rat osteoarthritis model induced by anterior cruciate ligament transection [83]. Mechanistically, TLR7 activation in DRG neurons induces inward currents via TRPA1, triggers action potentials [84], and enhances neuronal excitability by blocking multiple potassium channels [85]. Despite these extensive studies, the cellular and molecular mechanisms of TLR7 signaling in the spinal dorsal horn remain poorly understood. Our study demonstrates that TLR7 is specifically expressed in spinal microglia, but not in astrocytes or neurons (Figure 2), consistent with observations in the forebrain [29,30]. Activation of spinal microglial TLR7 induces both microglial and astrocytic activation, increases IL-1β and IL-18 production, and elevates Cav2.2 protein expression (Figure 5B) as well as neuronal activity (Figure 4) in the spinal dorsal horn. This is consistent with and reminiscent of our previous findings showing that astrocytes were activated by IL-1β released from microglia following paclitaxel-induced activation of microglial TLR4 [64]. Furthermore, intrathecal administration of a TLR7 agonist enhanced hindlimb thermal sensitivity in control mice (Figure 3B), while intrathecal administration of a TLR7 antagonist alleviated hindlimb thermal hypersensitivity in lupus mice (Figure 3A). These findings indicate that TLR7 signaling in spinal microglia is critically involved in the central sensitization and pathogenesis of chronic pain induced by SLE.

### 4.3. Molecular and Synaptic Mechanisms Underlying the Genesis of Chronic Pain Induced by SLE

Glutamatergic synapses are the primary excitatory synapses in the CNS including the spinal cord, driving neuronal activation through ionotropic glutamate receptors on postsynaptic neurons. At the synaptic level, glutamate receptor activation is determined by three main factors: the amount of glutamate released from presynaptic terminals, the efficacy of postsynaptic glutamate receptors, and the rate of glutamate clearance from the synaptic cleft. Because extracellular glutamate is not metabolized, its clearance depends primarily on glial glutamate transporters [18,86]. At glutamatergic synapses between primary nociceptive afferents and superficial dorsal horn neurons—the first relay in the pain pathway—these three factors are altered in lupus mice with chronic pain. Our present study demonstrated that lupus mice have enhanced presynaptic glutamate release as well as increased postsynaptic glutamate receptor responsiveness in the superficial dorsal horn neurons (Figure 6), consistent with previous reports [17,21]. Furthermore, protein expression and activity of glial glutamate transporters are reduced in lupus mice [19]. The resulting decrease in glutamate uptake prolongs and enhances activation of AMPA and NMDA receptors [49,50].

Recent studies, including ours, have shown that IL-1β and IL-18 modulate spinal glutamatergic activity. IL-1β enhances presynaptic glutamate release [51,52] and postsynaptic AMPA and NMDA receptor activity [52,87,88], while reducing glial glutamate transporter function [19,47]. IL-1β facilitates glutamate release by enhancing presynaptic NMDA receptor function [52], while IL-18 promotes presynaptic glutamate release in the spinal dorsal horn [14]. In the present study, activation of TLR7 in the spinal dorsal horn increased both presynaptic glutamate release and postsynaptic ionotropic receptor function, as reflected by elevated mEPSC frequencies and amplitudes in superficial dorsal horn neurons (Figure 8). Spinal tissue incubated with a TLR7 agonist for 2.5 h also showed elevated IL-1β and IL-18 levels. Therefore, enhanced TLR7 signaling in lupus mice likely amplifies neuronal activity and promotes spinal central sensitization by increasing the production and release of IL-1β and IL-18. This interpretation is further supported by our finding that pharmacological blockade of TLR7 with ODN2088 attenuated both presynaptic glutamate release and postsynaptic AMPA receptor activity in superficial dorsal horn neurons of lupus mice (Figure 7).

Furthermore, our current study implicates the involvement of spinal Cav2.2 in the pathogenesis of chronic pain induced by lupus. These findings align with previous studies on animal models with neuropathic pain [41,42] and inflammatory pain [89], where enhanced activity of Cav2.2 in the spinal dorsal horn was found. Cav2.2 knockdown or pharmacological inhibition alleviates pain-like behaviors in animals with inflammation and nerve injury [90,91,92,93] and reduce glutamate release from primary nociceptive terminals in the spinal dorsal horn [94]. These studies highlight Cav2.2 as a common pathway for excessive glutamate release across different pain etiologies. Signaling pathways regulating Cav2.2 expression are not fully understood. Our study demonstrated that increased Cav2.2 protein expression in the spinal dorsal horn of lupus mice with chronic pain is associated with elevated TLR7 expression, and that activation of TLR7 with a TLR7 agonist in the normal spinal cord upregulates Cav2.2 (Figure 5). These findings suggest that TLR7 activation acts as a trigger to enhance Cav2.2 function.

### 4.4. Role of Microglia in the Pathogenesis of Chronic Pain in Females

The role of microglia in the genesis of pathological pain across sexes remains controversial [95]. Some studies report that intrathecal injection of the microglial inhibitor minocycline reverses mechanical allodynia induced by spared nerve injury or CFA-induced inflammatory pain in male mice, but not in females [96]. Similarly, activation of spinal TLR4 via intrathecal lipopolysaccharide induces mechanical allodynia in males but not females [97]. Conversely, evidence also supports a critical role for microglia in female pain. Depletion of microglia and macrophages in the CNS and dorsal root ganglia abolishes mechanical and thermal hypersensitivity induced by spinal nerve transection in both sexes [98]. Gene knockdown of TLR4 prevents and reverses bone cancer pain in female rats [99]. Neutralizing the endogenous spinal TLR4 ligand HMGB1 with antibodies [100] or suppressing microglial activation [101] reverses mechanical hypersensitivity in female mice with collagen antibody-induced arthritis. Our recent studies provide behavioral, molecular, and electrophysiological evidence that dysfunctional microglia in the spinal dorsal horn contribute to the development of thermal hyperalgesia in female lupus mice. Blocking microglial M-CSF1 receptors alleviates thermal hyperalgesia and improves glial glutamate transporter activity in the dorsal horn [19]. Pharmacological activation of the Gi protein-coupled receptor GPR109A, expressed in microglia, markedly reduces thermal hyperalgesia in lupus mice without affecting normal sensory perception [17]. Activation of spinal microglial GPR109A suppresses p38 MAPK signaling and diminishes glutamatergic synaptic activity by decreasing IL-18 and IL-1β production in the dorsal horn of female lupus mice with chronic pain [17]. Our current study further supports the involvement of microglia in thermal hyperalgesia in female lupus mice. Activation of microglial TLR7 in the spinal dorsal horn of female mice induces microglial and astrocytic activation, enhances p38 MAPK signaling, increases IL-18 and IL-1β production, augments spinal glutamatergic synaptic activity, and results in heightened thermal sensitivity in the hind paw. These findings suggest that the contribution of microglia to pathological pain in females may depend on the underlying etiology, sensory modality, or signaling pathways involved, highlighting the need for further investigation.

Microglial and astrocytic activation [67,68,102], along with p38 MAPK [103,104], IL-18 [103,105], and IL-1β [52,64,105] signaling, play critical roles in the development of pathological pain induced by nerve injury, cancer, and inflammation in male animals. Given that these cellular and molecular alterations were also observed in our current study using female lupus mice, it is conceivable that our findings may likewise be applicable to male animals.

## 5. Conclusions

Our present study demonstrates that spinal microglial TLR7 signaling plays a critical role in promoting central sensitization and chronic pain by enhancing the expression of Cav2.2, IL-1β, and IL-18, as well as glutamatergic synaptic activity in the spinal dorsal horn. These findings not only deepen our understanding of the mechanisms underlying lupus-associated pain, but also suggest that targeting TLR7 or its downstream effectors may represent a promising strategy to alleviate chronic pain associated with SLE.

## Data Availability

The data that support the findings of this study are available from the corresponding author upon reasonable request.

## Supplementary Materials

The following supporting information can be downloaded at: https://www.mdpi.com/article/10.3390/cells15010020/s1, Figure S1: TLR7 protein was detected in microglia within the spinal dorsal horn of *MRL* control mice. Fluorescent images were captured from the dorsal region of the spinal dorsal horn in 16-week-old MRL control mice, with the inset showing an enlarged view of the area indicated by the rectangular box. In the spinal slices, microglia, astrocytes, and neurons were labeled green using Iba1, GFAP, and NeuN antibodies, respectively, while TLR7 was labeled in red. Colocalization of TLR7 with the respective cell markers is shown on the right.
