# Supplementary material for: Spinal Microglial TLR7 Activation Drives Hyperalgesia in a Lupus Mouse Model via Upregulation of IL-1β, IL-18, and Cav2.2 and Enhanced Glutamatergic Synaptic Activity
Source: Cells. 2025 Dec 22;15(1):20. doi: 10.3390/cells15010020 (PMC12785760; doi:10.3390/cells15010020)
Supplement: Supplementary file 1 [file cells-15-00020-s001.zip › cells-4026806-supplementary.pdf]

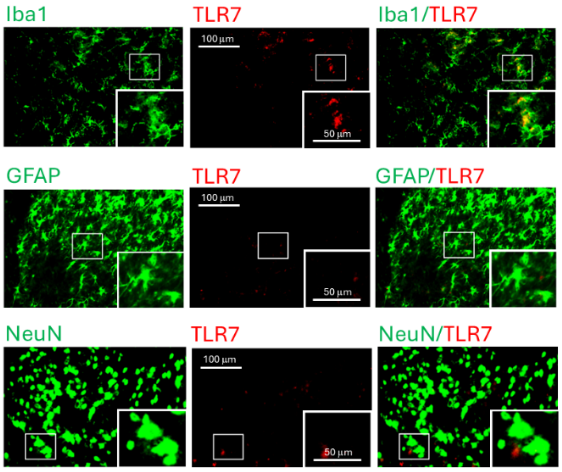

Figure S1. *TLR7 protein was detected in microglia within the spinal dorsal horn of MRL control mice.* Fluorescent images were captured from the dorsal region of the spinal dorsal horn in 16-week-old MRL control mice, with the inset showing an enlarged view of the area indicated by the rectangular box. In the spinal slices, microglia, astrocytes, and neurons were labeled green using Iba1, GFAP, and NeuN antibodies, respectively, while TLR7 was labeled in red. Colocalization of TLR7 with the respective cell markers is shown on the right.
